# PSYCHOSOCIAL CORRELATES OF PREVENTION, CARE, AND WELL-BEING AMONG OLDER WOMEN LIVING WITH HIV

**DOI:** 10.1093/geroni/igad104.0445

**Published:** 2023-12-21

**Authors:** Thi Vu, Anna Rubtsova

## Abstract

There are over 4.2 million older adults (ages 50+ years) living with human immunodeficiency virus (HIV) globally. Older women continue to make up a significant proportion of this population. Advances in antiretroviral therapy treatments have allowed HIV+ individuals to live longer, healthier lives. However, compared to all persons living with HIV, women have lower viral suppression rates and are less likely to be retained in care. While older women living with HIV (WLWH) face unique challenges with HIV prevention and care, such as caregiving responsibilities, gender discrimination, and ageism, there is a dearth of research focusing on their experiences. As the population of older WLWH continues to increase, understanding barriers and facilitators to HIV prevention and care among this population is a public health priority. This symposium will provide insight into psychosocial factors that influence HIV prevention, care, and well-being among older WLWH. Our first presentation highlights a qualitative study identifying sources of strength and concerns about aging with HIV among older women. Our second presentation examines social support networks and interpersonal strain in relation to loneliness among older WLWH. Our third presentation highlights the impact of patient-provider communication regarding HIV/AIDs on sexual health communication between older WLWH and their partners. Our fourth presentation examines mental health vulnerabilities and strengths of older WLWH during the humanitarian crisis in Ukraine. Discussant Dr. Anna Rubtsova will contextualize these findings and offer suggestions for future research to enhance well-being of older women across the continuum of HIV prevention and care.

